# Book Review: Introduction to Neuroimaging Analysis

**DOI:** 10.3389/fnins.2018.00674

**Published:** 2018-09-26

**Authors:** Peter Sörös, Karsten Witt

**Affiliations:** Neurology, School of Medicine and Health Sciences, and Research Center Neurosensory Science, University of Oldenburg, Oldenburg, Germany

**Keywords:** magnetic resonance imaging, fMRI, DTI, data analysis, general linear model

Magnetic resonance imaging (MRI) has become an essential research tool in human neuroscience, and MRI data analysis is a critical skill for students and researchers in the field. Learning—and teaching—the fundamentals of MRI data analysis is tricky, time-consuming and sometimes frustrating. Quantitative analyses of structural and functional MRI depend on knowledge in many disparate areas, such as MR physics, statistics. and computer programming, but also neurophysiology and neuroanatomy. Most of all, MRI data analysis is a practical skill and requires the knowledge of one of the major analysis software packages.

A new book by Jenkinson and Chappell, both at the University of Oxford and experienced teachers of MRI analysis, aims to instruct students and researchers who are new to the field of neuroimaging research.

Chapter 1 of *Introduction to Neuroimaging Analysis* provides a brief overview of the main MRI modalities, walks the reader through the several steps of a first, imaginary neuroimaging study and gives a concise introduction to MR physics and scanner hardware. Chapter 2 brings a more in-depth description of MRI modalities, primarily structural, functional, and diffusion MRI. The rest of the book discusses quantitative techniques of MRI analysis. Chapter 3, providing a concise and clear summary of structural, functional, and diffusion imaging pipelines, is the heart of the book. If you are a newbie to MRI analysis, you would want to read this chapter, and read it several times to absorb the many concepts introduced here. Additional chapters on brain extraction, registration as well as motion and distortion correction present a comprehensive discussion of major steps in data preprocessing. Finally, chapter 7 illustrates a more advanced method, surface-based analysis of structural and functional data.

The book is accompanied by additional chapters on the *Neuroimaging Primers* website. The *MRI Physics for Neuroimaging* appendix provides a short, non-technical overview of the basics of MR physics. The *General Linear Model for Neuroimaging* appendix is a very well-written and illustrated introduction to the centerpiece of statistical analysis of MRI.

The unique feature of this book is the use of Example Boxes that provide practical knowledge and skills for MRI analysis. Many of these boxes are linked to separate web pages on the *Neuroimaging Primers* website. These web pages start with instructions how to install and run FSL, the authors' favorite neuroimaging software package (of which Mark Jenkinson is principal developer). FSL is a software package that includes programs for the analysis of structural MRI, task-based and resting state functional MRI and diffusion tensor imaging and that runs on Unix systems (such as Mac OS), on Linux machines and inside a virtual Linux machine on Windows PCs.

Additional web pages cover, for example, analysis of structural MRI (such as tissue-type segmentation), diffusion tensor imaging (such as tractography) and task-based functional MRI. These web pages act as short online tutorials; they contain MRI data sets for download and demonstrate how to analyze the data in FSL and visualize the results using FSL's image viewer FSLeyes. We ran several of these tutorials and all worked well. The data downloaded from the tutorial web page not only include the original data set, but also FSL's results folder. This allows you to check and comprehend the results of every single analysis introduced on the web pages without running the analysis yourself or even installing FSL. These tutorials are ideal for homework, they are written with the student in mind and easy to follow.

Running FSL through the graphical user interface requires only rudimentary UNIX skills, which are introduced on the “Getting Started” web page. Those who long for additional UNIX proficiency simply take the link to the FSL course web page, containing Introduction to UNIX videos and a UNIX handout. To sum up, if you are FSL-inclined and teaching an introductory course on MRI data analysis covering the basics of structural MRI, fMRI, and DTI, this book is everything you need. If the main focus of your course is task-based functional MRI, you may need more detail and you may want to add recent review articles or chapters of the established fMRI textbooks (see below) to the reading list.

If you prefer a different software package, let's say AFNI, Brainvoyager or SPM, the new textbook by Jenkinson and Chappell is still a very helpful resource. The main text of the book and the online appendices are software-independent. The original and the analyzed data sets on the web pages can be inspected by any nifti file viewer. The web page on MRI artifacts, e.g., offers 25 MRI data sets covering various artifacts. Of course, the web pages describe the commands FSL uses to perform a certain analysis step. If you would like your student to learn a non-FSL package through these web pages, you would need to provide the necessary commands and code.

Jenkinson and Chappell's book is the most recent of several publications on neuroimaging methods. The textbook by Huettel, Song and McCarthy, *Functional Magnetic Resonance Imaging*, now in its 3rd edition, has gained a wide readership among students and is used in numerous university courses (Huettel et al., 2014). The textbook by Huettel et al. is useful for undergraduate and graduate students who expect a more detailed, narrative account and who are primarily interested in task-based fMRI. The *Handbook of Functional MRI Data Analysis* by Poldrack, Mumford, and Nichols presents a more concise and slightly more technical discussion of fMRI analysis techniques (Poldrack et al., 2011). If your focus is on resting state fMRI, you may turn to *Introduction to Resting State fMRI Functional Connectivity* by Bijsterbosch, Smith and Beckmann, a recent addition to the *Oxford Neuroimaging Primers* series (Bijsterbosch et al., 2018).

Taken together, Jenkinson and Chappell have crafted an easily accessible and highly readable introduction to the analysis of structural MRI, functional MRI and diffusion tensor imaging. With the online tutorials, tightly connected with the main text, the reader will obtain useful hands-on methodological experience. The combination of main text and online tutorial makes this book an ideal companion for students and researchers who plan to undergo their first steps in structural, functional, or diffusion tensor imaging analysis.

Hopefully, this book will find many readers and see a 2nd edition in the near future. In the next edition, a few points should be revised. As the general linear model has become so important for MRI analysis, the authors may consider including their online chapter on the general linear model in the 2nd edition of the printed book. If necessary, Appendix A, Introduction to Brain Anatomy, might be removed and made online-only. Moreover, the online tutorial on *Physiological noise modeling*, announced but not available yet, would be very much appreciated. Finally, there is one crucial concept in neuroimaging analysis that we missed in this, otherwise excellent, book: a detailed description of methods for multiple comparison correction. This topic has been widely discussed, even in popular media, following the publication of Eklund et al.'s paper in 2016 (Eklund et al., 2016).

## Author contributions

All authors listed have made a substantial, direct and intellectual contribution to the work, and approved it for publication.

### Conflict of interest statement

The authors declare that the research was conducted in the absence of any commercial or financial relationships that could be construed as a potential conflict of interest.
